# Supplementary material for: Joint associations between objectively measured physical activity volume and intensity with body fatness: the Fenland study
Source: Int J Obes (Lond). 2021 Sep 30;46(1):169–77. doi: 10.1038/s41366-021-00970-8 (PMC8748201; doi:10.1038/s41366-021-00970-8)
Supplement: Supplementary file 1 — Supplementary Materials Legend [file 41366_2021_970_MOESM1_ESM.docx]

**Supplement Legend:**

**Supplementary Table 1:** Isocaloric substitution of physical activity energy expenditure and fat mass index.

**Supplementary Table 2:** Isocaloric substitution of physical activity energy expenditure and body fat percentage using alternative intensity thresholds (MPA > 4 METs, VPA > 7METs).

**Supplementary Table 3:** Isocaloric substitution of physical activity energy expenditure and body fat percentage by tertile of PAEE.

**Supplementary Table 4:** Relationship between isocaloric z1 ILR coordinate and body fat percentage.

**Supplementary Table 5:** Relationship between isocaloric pairwise ILR coordinates and body fat percentage.

**Supplementary Table 6:** Relationship between isocaloric z1 ILR coordinate and body fat percentage (MPA > 4MET, VPA > 7METs).

**Supplementary Table 7:** Relationship between isocaloric z1 ILR coordinate and body fat percentage by tertile of PAEE.

**Supplementary Table 8:** Isotemporal substitution of physical activity and body fat percentage.

**Supplementary Table 9:** Relationship between isotemporal z1 ILR coordinate and body fat percentage.

**Supplementary Table 10:** Relationship between isotemporal pairwise ILR coordinates and body fat percentage.

**Figure S1:** Pairwise and linear isocaloric reallocation of PAEE from one intensity to another. The top panel shows the results of pairwise compositional analysis, whereas the bottom panel shows linear substitution analysis. Both models estimate the difference in body fat percentage per 1% of PAEE reallocated. Group PAEE values are mean (SD). SS = 0-1.5 METs, LPA = 1.5-3 METs, MPA = 3-6 METs, VPA >6 METs.

**Figure S2:** Estimated difference in body fat % associated with the isocaloric reallocation of PAEE to different intensities and box plots of the distribution of the PAEE composition, stratified by sex. Intensity thresholds redefined as SS = 0-1.5 METs, LPA = 1.5-4 METs, MPA = 4-7 METs, VPA >7 METs.

The top panel shows the relative estimated difference in body fat percentage associated with an isocaloric reallocation of energy proportionately from all behaviours to the intensity of interest, as modelled by compositional data analysis. The origin (x=0,y=0) represents no change in the intensity composition of PAEE from the mean composition of the group of interest (women and men). The bottom panel illustrates the relative size of each reservoir of energy across women and men. Group PAEE values are mean (SD).

**Figure S3:** Estimated difference in body fat percentage associated with the isotemporal reallocation of time to different intensities, and box plots of the distribution of time by intensities, stratified by sex.

The top panel shows the relative estimated difference in body fat percentage associated with the reallocation of time proportionately from all behaviours to the intensity of interest, as modelled by compositional data analysis. The origin (x=0,y=0) represents no change in the intensity composition of time from the mean composition of the group of interest (women and men). The bottom panel illustrates the relative size of each reservoir of time across women and men. SS = 0-1.5 METs, LPA = 1.5-3 METs, MPA = 3-6 METs, VPA >6 METs.

**Figure S4:** Pairwise and linear isotemporal reallocation of time from one intensity to another. The top panel shows the results of pairwise compositional analysis, whereas the bottom panel shows linear substitution analysis. Both models estimate the percentage difference in body fat % per minute per day reallocated. SS = 0-1.5 METs, LPA = 1.5-3 METs, MPA = 3-6 METs, VPA >6 METs.
