# Supplementary material for: Joint associations between objectively measured physical activity volume and intensity with body fatness: the Fenland study
Source: Int J Obes (Lond). 2021 Sep 30;46(1):169–77. doi: 10.1038/s41366-021-00970-8 (PMC8748201; doi:10.1038/s41366-021-00970-8)
Supplement: Supplementary file 2 — Supplemental Figure 1 [file 41366_2021_970_MOESM2_ESM.pptx]

## Slide 1
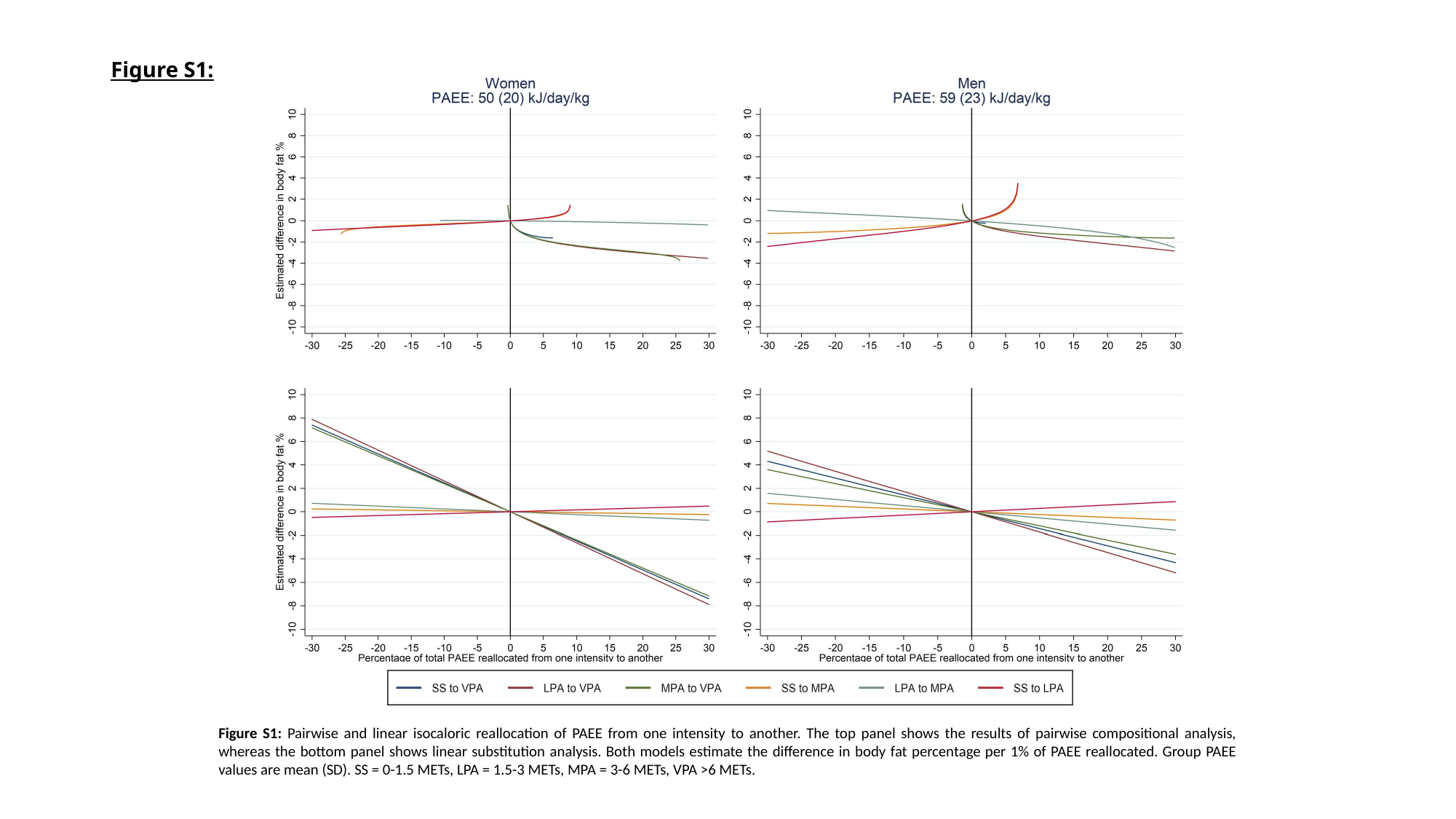

Figure S1:
Figure S1: Pairwise and linear isocaloric reallocation of PAEE from one intensity to another. The top panel shows the results of pairwise compositional analysis, whereas the bottom panel shows linear substitution analysis. Both models estimate the difference in body fat percentage per 1% of PAEE reallocated. Group PAEE values are mean (SD). SS = 0-1.5 METs, LPA = 1.5-3 METs, MPA = 3-6 METs, VPA >6 METs.
