# Supplementary material for: Joint associations between objectively measured physical activity volume and intensity with body fatness: the Fenland study
Source: Int J Obes (Lond). 2021 Sep 30;46(1):169–77. doi: 10.1038/s41366-021-00970-8 (PMC8748201; doi:10.1038/s41366-021-00970-8)
Supplement: Supplementary file 3 — Supplemental Figure 2 [file 41366_2021_970_MOESM3_ESM.pptx]

## Slide 1
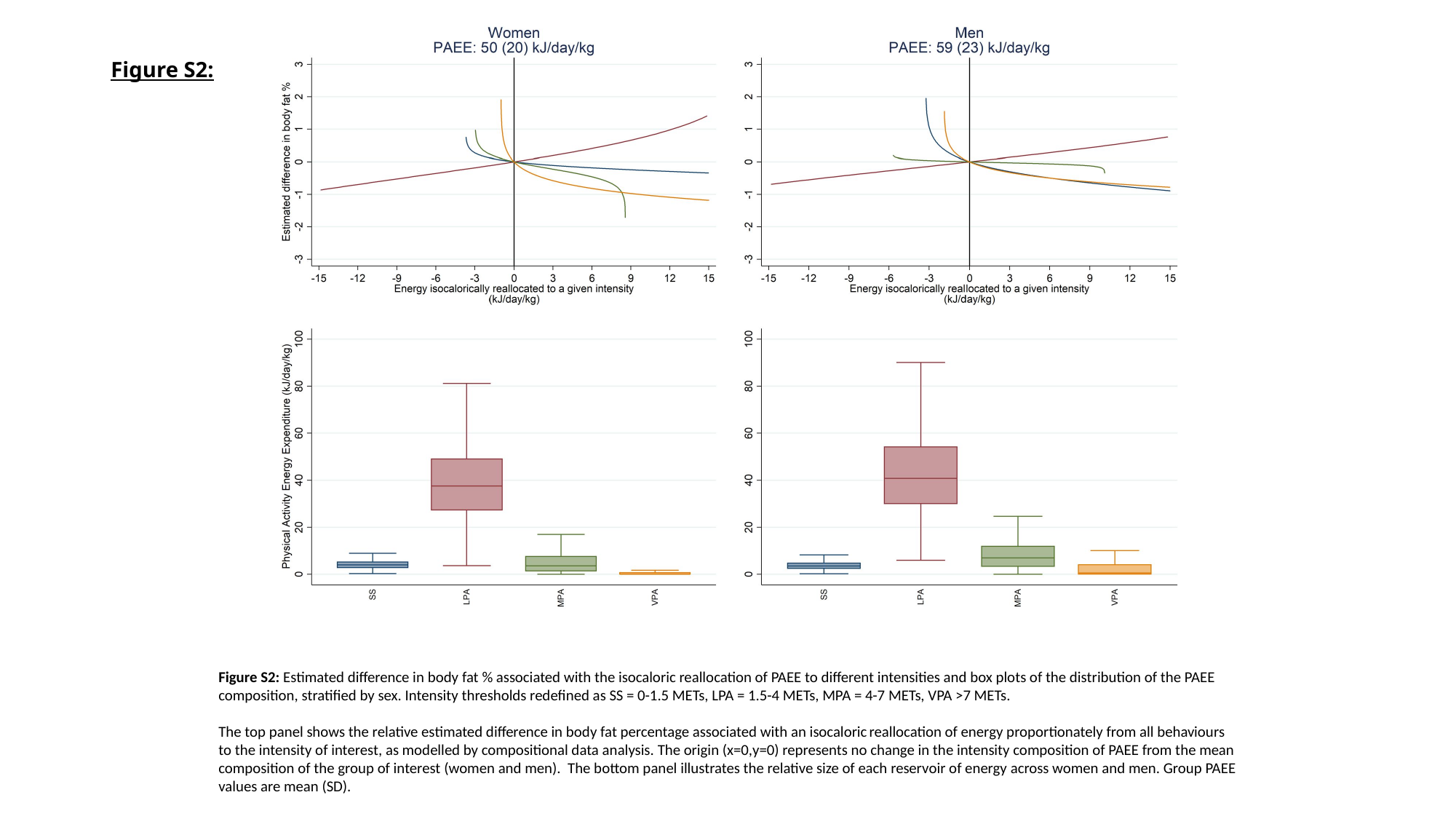

Figure S2:
Figure S2: Estimated difference in body fat % associated with the isocaloric reallocation of PAEE to different intensities and box plots of the distribution of the PAEE composition, stratified by sex. Intensity thresholds redefined as SS = 0-1.5 METs, LPA = 1.5-4 METs, MPA = 4-7 METs, VPA >7 METs.
The top panel shows the relative estimated difference in body fat percentage associated with an isocaloric reallocation of energy proportionately from all behaviours to the intensity of interest, as modelled by compositional data analysis. The origin (x=0,y=0) represents no change in the intensity composition of PAEE from the mean composition of the group of interest (women and men). The bottom panel illustrates the relative size of each reservoir of energy across women and men. Group PAEE values are mean (SD).
