# Supplementary material for: Joint associations between objectively measured physical activity volume and intensity with body fatness: the Fenland study
Source: Int J Obes (Lond). 2021 Sep 30;46(1):169–77. doi: 10.1038/s41366-021-00970-8 (PMC8748201; doi:10.1038/s41366-021-00970-8)
Supplement: Supplementary file 4 — Supplemental Figure 3 [file 41366_2021_970_MOESM4_ESM.pptx]

## Slide 1
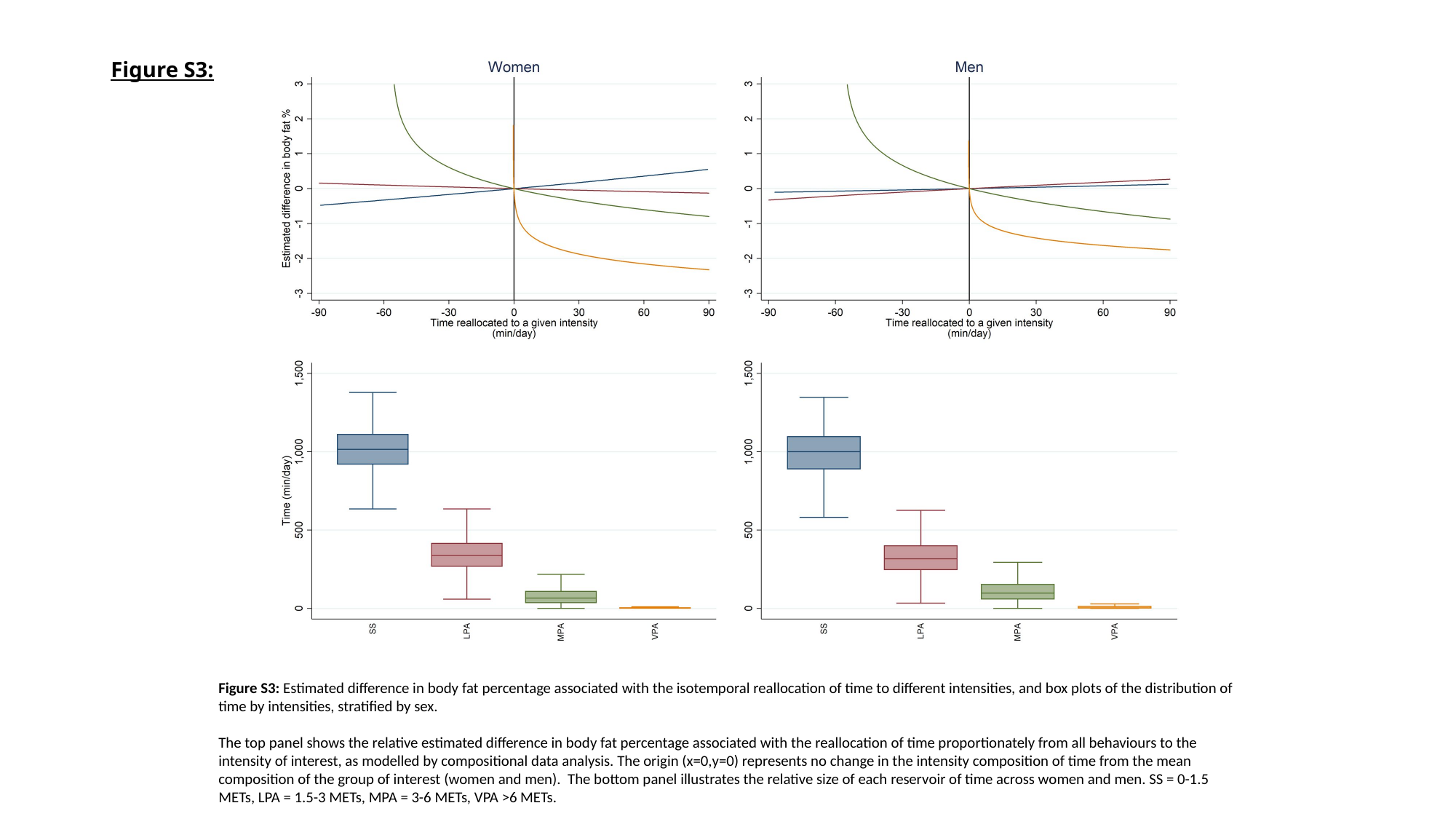

Figure S3:
Figure S3: Estimated difference in body fat percentage associated with the isotemporal reallocation of time to different intensities, and box plots of the distribution of time by intensities, stratified by sex.
The top panel shows the relative estimated difference in body fat percentage associated with the reallocation of time proportionately from all behaviours to the intensity of interest, as modelled by compositional data analysis. The origin (x=0,y=0) represents no change in the intensity composition of time from the mean composition of the group of interest (women and men). The bottom panel illustrates the relative size of each reservoir of time across women and men. SS = 0-1.5 METs, LPA = 1.5-3 METs, MPA = 3-6 METs, VPA >6 METs.
