# Supplementary material for: Joint associations between objectively measured physical activity volume and intensity with body fatness: the Fenland study
Source: Int J Obes (Lond). 2021 Sep 30;46(1):169–77. doi: 10.1038/s41366-021-00970-8 (PMC8748201; doi:10.1038/s41366-021-00970-8)
Supplement: Supplementary file 5 — Supplemental Figure 4 [file 41366_2021_970_MOESM5_ESM.pptx]

## Slide 1
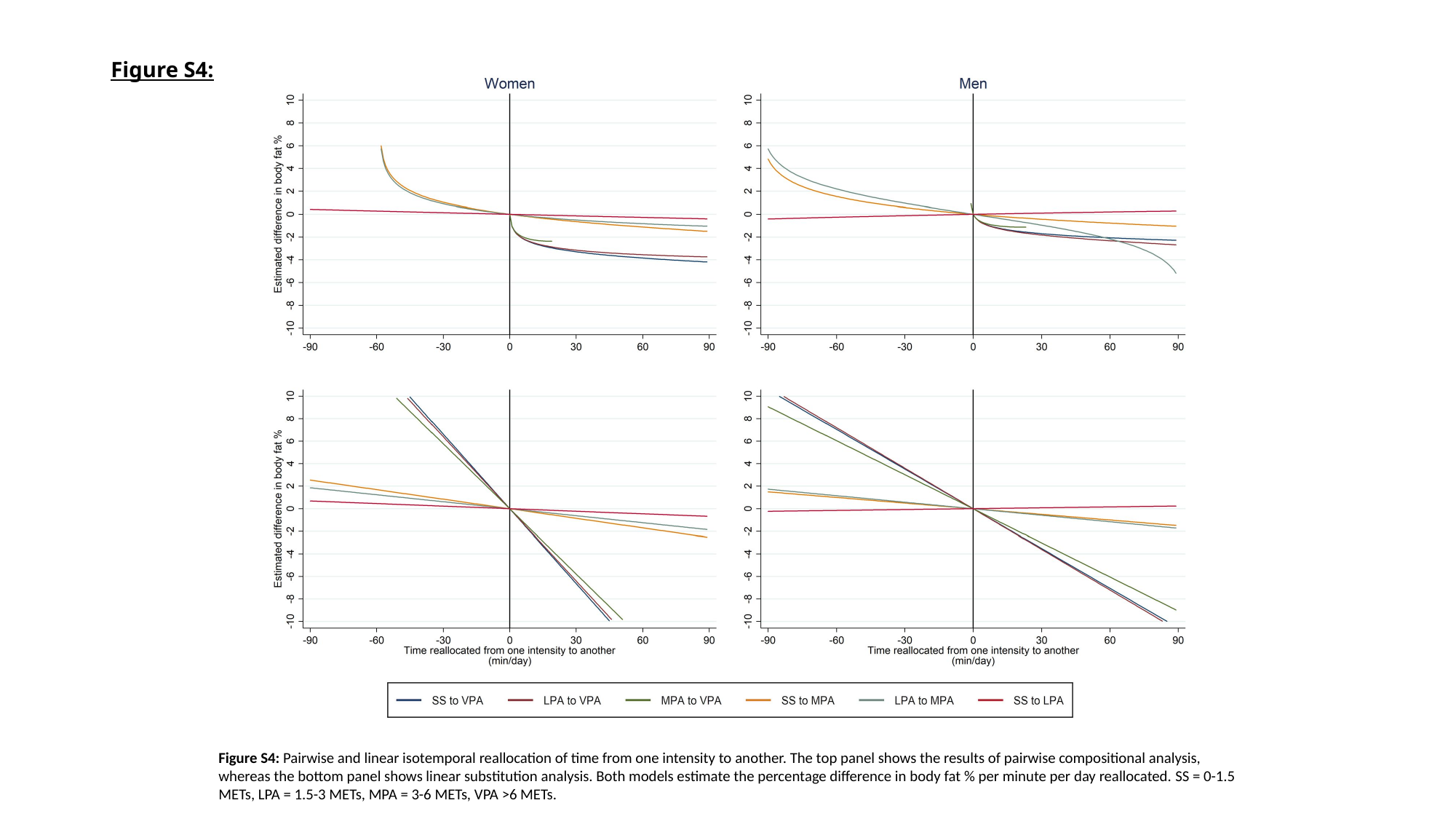

Figure S4:
Figure S4: Pairwise and linear isotemporal reallocation of time from one intensity to another. The top panel shows the results of pairwise compositional analysis, whereas the bottom panel shows linear substitution analysis. Both models estimate the percentage difference in body fat % per minute per day reallocated. SS = 0-1.5 METs, LPA = 1.5-3 METs, MPA = 3-6 METs, VPA >6 METs.
