# Supplementary material for: Joint associations between objectively measured physical activity volume and intensity with body fatness: the Fenland study
Source: Int J Obes (Lond). 2021 Sep 30;46(1):169–77. doi: 10.1038/s41366-021-00970-8 (PMC8748201; doi:10.1038/s41366-021-00970-8)
Supplement: Supplementary file 6 — Supplemental Table 1 [file 41366_2021_970_MOESM6_ESM.pdf]

**Supplementary Table 1: Isocaloric substitution of physical activity energy expenditure and fat mass index**

| | Substituted from SS | Substituted from LPA | Substituted from MPA | Substituted from VPA |
|---|---|---|---|---|
| Women n = 6148 | | | | |
| PAEE (kJ/day/kg) | | -0.05***<br>(-0.06 ; -0.04) | | |
| Substituted to SS (% of PAEE) | *Dropped* | 0.02**<br>(0.00 ; 0.03) | 0.04***<br>(0.02 ; 0.05) | 0.11***<br>(0.09 ; 0.13) |
| Substituted to LPA (% of PAEE) | -0.02**<br>(-0.03 ; -0.00) | *Dropped* | 0.02***<br>(0.01 ; 0.03) | 0.10***<br>(0.08 ; 0.11) |
| Substituted to MPA (% of PAEE) | -0.04***<br>(-0.05 ; -0.02) | -0.02***<br>(-0.03 ; -0.01) | *Dropped* | 0.08***<br>(0.06 ; 0.10) |
| Substituted to VPA (% of PAEE) | -0.11***<br>(-0.13 ; -0.09) | -0.09***<br>(-0.11 ; -0.08) | -0.08***<br>(-0.10 ; -0.06) | *Dropped* |
| Men n = 5320 | | | | |
| PAEE (kJ/day/kg) | | -0.02***<br>(-0.02 ; -0.01) | | |
| Substituted to SS (% of PAEE) | *Dropped* | -0.01<br>(-0.02 ; 0.01) | 0.02***<br>(0.01 ; 0.04) | 0.06***<br>(0.04 ; 0.07) |
| Substituted to LPA (% of PAEE) | 0.01<br>(-0.01 ; 0.02) | *Dropped* | 0.03***<br>(0.02 ; 0.03) | 0.06***<br>(0.05 ; 0.07) |
| Substituted to MPA (% of PAEE) | -0.02***<br>(-0.04 ; -0.01) | -0.03***<br>(-0.03 ; -0.02) | *Dropped* | 0.04***<br>(0.03 ; 0.04) |
| Substituted to VPA (% of PAEE) | -0.06***<br>(-0.07 ; -0.04) | -0.06***<br>(-0.07 ; -0.05) | -0.04***<br>(-0.04 ; -0.03) | *Dropped* |

Note: Data are beta coefficients (95% c.i.). The unit for the PAEE result is difference in FMI per 1kj/kg/day difference in PAEE. The unit for substitution results is difference in FMI per 1% of PAEE substituted. SS = Sedentary or sleep, LPA = Light physical activity, MPA = Moderate physical activity, VPA = Vigorous physical activity.
*** p<0.01, ** p<0.05, * p<0.1
