# Supplementary material for: Joint associations between objectively measured physical activity volume and intensity with body fatness: the Fenland study
Source: Int J Obes (Lond). 2021 Sep 30;46(1):169–77. doi: 10.1038/s41366-021-00970-8 (PMC8748201; doi:10.1038/s41366-021-00970-8)
Supplement: Supplementary file 7 — Supplemental Table 2 [file 41366_2021_970_MOESM7_ESM.pdf]

Supplementary Table 2: Isocaloric substitution of physical activity energy expenditure and body fat percentage using alternative intensity thresholds (MPA > 4 METs, VPA > 7 METs)

Women n = 6148

PAEE (kJ/day/kg)	-0.13***<br>(-0.14 ; -0.11)

	Substituted from SS	Substituted from LPA	Substituted from MPA	Substituted from VPA
Substituted to SS (% of PAEE)	*Dropped*	-0.01<br>(-0.04 ; 0.02)	0.06***<br>(0.02 ; 0.10)	0.29***<br>(0.24 ; 0.34)
Substituted to LPA (% of PAEE)	0.01<br>(-0.02 ; 0.04)	*Dropped*	0.07***<br>(0.04 ; 0.09)	0.30***<br>(0.26 ; 0.34)
Substituted to MPA (% of PAEE)	-0.06***<br>(-0.10 ; -0.02)	-0.07***<br>(-0.09 ; -0.04)	*Dropped*	0.23***<br>(0.18 ; 0.28)
Substituted to VPA (% of PAEE)	-0.29***<br>(-0.34 ; -0.24)	-0.30***<br>(-0.34 ; -0.26)	-0.23***<br>(-0.28 ; -0.18)	*Dropped*

Men n = 5320

PAEE (kJ/day/kg)	-0.07***<br>(-0.08 ; -0.05)

	Substituted from SS	Substituted from LPA	Substituted from MPA	Substituted from VPA
Substituted to SS (% of PAEE)	*Dropped*	-0.01<br>(-0.04 ; 0.03)	0.02<br>(-0.02 ; 0.06)	0.18***<br>(0.13 ; 0.22)
Substituted to LPA (% of PAEE)	0.01<br>(-0.03 ; 0.04)	*Dropped*	0.03***<br>(0.01 ; 0.05)	0.18***<br>(0.16 ; 0.21)
Substituted to MPA (% of PAEE)	-0.02<br>(-0.06 ; 0.02)	-0.03***<br>(-0.05 ; -0.01)	*Dropped*	0.16***<br>(0.12 ; 0.19)
Substituted to VPA (% of PAEE)	-0.18***<br>(-0.22 ; -0.13)	-0.18***<br>(-0.21 ; -0.16)	-0.15***<br>(-0.18 ; -0.12)	*Dropped*

Note: Data are beta coefficients (95% c.i.). The unit for the PAEE result is difference in body fat percentage per 1kj/kg/day difference in PAEE. The unit for substitution results is difference in body fat percentage per 1% of PAEE substituted. SS = Sedentary or sleep, LPA = Light physical activity, MPA = Moderate physical activity, VPA = Vigorous physical activity.

*** p<0.01, ** p<0.05, * p<0.1
