# Supplementary material for: Joint associations between objectively measured physical activity volume and intensity with body fatness: the Fenland study
Source: Int J Obes (Lond). 2021 Sep 30;46(1):169–77. doi: 10.1038/s41366-021-00970-8 (PMC8748201; doi:10.1038/s41366-021-00970-8)
Supplement: Supplementary file 8 — Supplemental Table 3 Revised [file 41366_2021_970_MOESM8_ESM.pdf]

Supplementary Table 3: Isocaloric substitution of physical activity energy expenditure and body fat percentage by tertile of PAEE.

Women

Tertile 1

Women n = 2050

PAEE (kJ/day/kg)	-0.27***			
	(-0.34 ; -0.21)			
	Substituted from SS	Substituted from LPA	Substituted from MPA	Substituted from VPA
Substituted to SS (% of PAEE)	*Dropped*	-0.11***	-0.06**	0.27***
		(-0.15 ; -0.06)	(-0.11 ; -0.01)	(0.16 ; 0.38)
Substituted to LPA (% of PAEE)	0.11***	*Dropped*	0.05**	0.38***
	(0.06 ; 0.15)		(0.01 ; 0.08)	(0.27 ; 0.48)
Substituted to MPA (% of PAEE)	0.06**	-0.05**	*Dropped*	0.33***
	(0.01 ; 0.11)	(-0.08 ; -0.01)		(0.22 ; 0.44)
Substituted to VPA (% of PAEE)	-0.27***	-0.38***	-0.33***	*Dropped*
	(-0.38 ; -0.16)	(-0.48 ; -0.27)	(-0.44 ; -0.22)	

Tertile 2

Women n = 2049

PAEE (kJ/day/kg)	-0.11***			
	(-0.18 ; -0.04)			
	Substituted from SS	Substituted from LPA	Substituted from MPA	Substituted from VPA
Substituted to SS (% of PAEE)	*Dropped*	-0.06	-0.01	0.20***
		(-0.16 ; 0.04)	(-0.11 ; 0.09)	(0.09 ; 0.32)
Substituted to LPA (% of PAEE)	0.06	*Dropped*	0.05***	0.26***
	(-0.04 ; 0.16)		(0.01 ; 0.08)	(0.20 ; 0.32)
Substituted to MPA (% of PAEE)	0.01	-0.05***	*Dropped*	0.22***
	(-0.08 ; 0.11)	(-0.08 ; -0.01)		(0.15 ; 0.28)
Substituted to VPA (% of PAEE)	-0.20***	-0.26***	-0.21***	*Dropped*
	(-0.32 ; -0.09)	(-0.32 ; -0.20)	(-0.28 ; -0.15)	

Tertile 3

Women n = 2049

PAEE (kJ/day/kg)	-0.12***			
	(-0.15 ; -0.09)			
	Substituted from SS	Substituted from LPA	Substituted from MPA	Substituted from VPA
Substituted to SS (% of PAEE)	*Dropped*	-0.08	-0.09	0.15*
		(-0.24 ; 0.07)	(-0.24 ; 0.07)	(-0.01 ; 0.31)
Substituted to LPA (% of PAEE)	0.08	*Dropped*	-0.00	0.24***
	(-0.07 ; 0.24)		(-0.04 ; 0.03)	(0.19 ; 0.28)
Substituted to MPA (% of PAEE)	0.09	0.00	*Dropped*	0.24***
	(-0.07 ; 0.25)	(-0.03 ; 0.04)		(0.19 ; 0.29)
Substituted to VPA (% of PAEE)	-0.15*	-0.24***	-0.24***	*Dropped*
	(-0.31 ; 0.01)	(-0.28 ; -0.19)	(-0.29 ; -0.19)	

Men

Tertile 1

Men n = 1774

PAEE (kJ/day/kg)	-0.09***			
	(-0.14 ; -0.04)			
	Substituted from SS	Substituted from LPA	Substituted from MPA	Substituted from VPA
Substituted to SS (% of PAEE)	*Dropped*	-0.00	0.02	0.15***
		(-0.06 ; 0.05)	(-0.03 ; 0.08)	(0.09 ; 0.22)
Substituted to LPA (% of PAEE)	0.00	*Dropped*	0.03	0.16***
	(-0.05 ; 0.06)		(-0.01 ; 0.06)	(0.11 ; 0.20)
Substituted to MPA (% of PAEE)	-0.02	-0.03	*Dropped*	0.13***
	(-0.07 ; 0.03)	(-0.06 ; 0.01)		(0.08 ; 0.18)
Substituted to VPA (% of PAEE)	-0.15***	-0.16***	-0.13***	*Dropped*
	(-0.22 ; -0.09)	(-0.20 ; -0.11)	(-0.18 ; -0.08)	

Tertile 2

Men n = 1773

PAEE (kJ/day/kg)	-0.06**			
	(-0.11 ; -0.00)			
	Substituted from SS	Substituted from LPA	Substituted from MPA	Substituted from VPA
Substituted to SS (% of PAEE)	*Dropped*	-0.08	-0.02	0.10*
		(-0.20 ; 0.03)	(-0.13 ; 0.09)	(-0.01 ; 0.21)
Substituted to LPA (% of PAEE)	0.09	*Dropped*	0.06***	0.18***
	(-0.03 ; 0.20)		(0.03 ; 0.09)	(0.15 ; 0.22)
Substituted to MPA (% of PAEE)	0.02	-0.06***	*Dropped*	0.12***
	(-0.09 ; 0.13)	(-0.09 ; -0.03)		(0.08 ; 0.16)
Substituted to VPA (% of PAEE)	-0.10*	-0.18***	-0.12***	*Dropped*
	(-0.21 ; 0.02)	(-0.22 ; -0.15)	(-0.16 ; -0.08)	

Tertile 3

Men n = 1773

PAEE (kJ/day/kg)	-0.08***			
	(-0.10 ; -0.05)			
	Substituted from SS	Substituted from LPA	Substituted from MPA	Substituted from VPA
Substituted to SS (% of PAEE)	*Dropped*	-0.55***	-0.48***	-0.37***
		(-0.72 ; -0.38)	(-0.65 ; -0.31)	(-0.54 ; -0.20)
Substituted to LPA (% of PAEE)	0.55***	*Dropped*	0.07***	0.18***
	(0.38 ; 0.72)		(0.04 ; 0.10)	(0.14 ; 0.22)
Substituted to MPA (% of PAEE)	0.48***	-0.07***	*Dropped*	0.11***
	(0.31 ; 0.66)	(-0.10 ; -0.04)		(0.07 ; 0.14)
Substituted to VPA (% of PAEE)	0.37***	-0.18***	-0.11***	*Dropped*
	(0.20 ; 0.55)	(-0.22 ; -0.14)	(-0.14 ; -0.07)	

Note: Data are beta coefficients (95% c.i.). The unit for the PAEE result is difference in body fat percentage per 1kj/kg/day difference in PAEE. The unit for substitution results is difference in body fat percentage per 1% of PAEE substituted. SS = Sedentary or sleep, LPA = Light physical activity, MPA = Moderate physical activity, VPA = Vigorous physical activity.
*** p<0.01, ** p<0.05, * p<0.1
