# Supplementary material for: Joint associations between objectively measured physical activity volume and intensity with body fatness: the Fenland study
Source: Int J Obes (Lond). 2021 Sep 30;46(1):169–77. doi: 10.1038/s41366-021-00970-8 (PMC8748201; doi:10.1038/s41366-021-00970-8)
Supplement: Supplementary file 9 — Supplemental Table 4 [file 41366_2021_970_MOESM9_ESM.pdf]

Supplementary Table 4: Relationship between isocaloric z1 ILR coordinate and body fat percentage

Women n = 6148	coef	se	tstat	pval
SS:remaining behaviours	-0.18*	(0.11)	-1.65	0.10
LPA:remaining behaviours	0.55***	(0.14)	4.02	0.00
MPA:remaining behaviours	0.11	(0.10)	1.07	0.29
VPA:remaining behaviours	-0.48***	(0.03)	-15.31	0.00
Total PAEE	-0.13***	(0.01)	-15.95	0.00

Men n = 5320	coef	se	tstat	pval
SS:remaining behaviours	-0.52***	(0.10)	-5.15	0.00
LPA:remaining behaviours	0.95***	(0.13)	7.27	0.00
MPA:remaining behaviours	-0.04	(0.12)	-0.38	0.71
VPA:remaining behaviours	-0.38***	(0.03)	-13.23	0.00
Total PAEE	-0.08***	(0.01)	-11.51	0.00

Note: Beta coefficients speak to direction and significance, but not magnitude of reallocation effect. SS = Sedentary or sleep, LPA = Light physical activity, MPA = Moderate physical activity, VPA = Vigorous physical activity.
*** p<0.01, ** p<0.05, * p<0.1
