# Supplementary material for: Joint associations between objectively measured physical activity volume and intensity with body fatness: the Fenland study
Source: Int J Obes (Lond). 2021 Sep 30;46(1):169–77. doi: 10.1038/s41366-021-00970-8 (PMC8748201; doi:10.1038/s41366-021-00970-8)
Supplement: Supplementary file 10 — Supplemental Table 5 [file 41366_2021_970_MOESM10_ESM.pdf]

Supplementary Table 5: Relationship between isocaloric pairwise ILR coordinates and body fat percentage

	Women n = 6148				Men = 5320			
	coef	se	tstat	pval	coef	se	tstat	pval
**Physical Activity Energy Expenditure**	-0.13***	(0.01)	-15.95	0.00	-0.08***	(0.01)	-11.51	0.00
**Composition of intensity (Full ILR coordinate set)**	-	-	-	0.00†	-	-	-	0.00†
**Pairwise reallocation - SS to LPA**								
$ilr_1 \propto$ ln(LPA & SS:MPA & VPA)	0.56***	(0.15)	3.73	0.00	0.64***	(0.17)	3.81	0.00
$ilr_2 \propto$ ln(LPA:SS)	0.78***	(0.24)	3.23	0.00	1.56***	(0.22)	7.18	0.00
$ilr_3 \propto$ ln(MPA:VPA)	0.63***	(0.12)	5.07	0.00	0.36***	(0.14)	2.66	0.01
**Pairwise reallocation - SS to MPA**								
$ilr_1 \propto$ ln(MPA & SS:LPA & VPA)	-0.10	(0.21)	-0.48	0.63	-0.85***	(0.20)	-4.17	0.00
$ilr_2 \propto$ ln(MPA:SS)	0.31*	(0.17)	1.85	0.06	0.50***	(0.18)	2.78	0.01
$ilr_3 \propto$ ln(LPA:VPA)	1.10***	(0.15)	7.47	0.00	1.41***	(0.14)	10.10	0.00
**Pairwise reallocation - SS to VPA**								
$ilr_1 \propto$ ln(VPA & SS:MPA & LPA)	-0.99***	(0.16)	-6.03	0.00	-1.36***	(0.16)	-8.72	0.00
$ilr_2 \propto$ ln(VPA:SS)	-0.32***	(0.12)	-2.60	0.01	0.14	(0.11)	1.28	0.20
$ilr_3 \propto$ ln(MPA:LPA)	-0.47**	(0.23)	-2.03	0.04	-1.05***	(0.24)	-4.41	0.00
**Pairwise reallocation - LPA to MPA**								
$ilr_1 \propto$ ln(MPA & LPA:SS & VPA)	0.99***	(0.16)	6.03	0.00	1.36***	(0.16)	8.72	0.00
$ilr_2 \propto$ ln(MPA:LPA)	-0.47**	(0.23)	-2.03	0.04	-1.05***	(0.24)	-4.41	0.00
$ilr_3 \propto$ ln(SS:VPA)	0.32***	(0.12)	2.60	0.01	-0.14	(0.11)	-1.28	0.20
**Pairwise reallocation - LPA to VPA**								
$ilr_1 \propto$ ln(VPA & LPA:SS & MPA)	0.10	(0.21)	0.48	0.63	0.85***	(0.20)	4.17	0.00
$ilr_2 \propto$ ln(VPA:LPA)	-1.10***	(0.15)	-7.47	0.00	-1.41***	(0.14)	-10.10	0.00
$ilr_3 \propto$ ln(SS:MPA)	-0.31*	(0.17)	-1.85	0.06	-0.50***	(0.18)	-2.78	0.01
**Pairwise reallocation - MPA to VPA**								
$ilr_1 \propto$ ln(MPA & VPA:SS & LPA)	-0.56***	(0.15)	-3.73	0.00	-0.64***	(0.17)	-3.81	0.00
$ilr_2 \propto$ ln(VPA:MPA)	-0.63***	(0.12)	-5.07	0.00	-0.36***	(0.14)	-2.66	0.01
$ilr_3 \propto$ *ln(SS:LPA)*	-0.78***	(0.24)	-3.23	0.00	-1.56***	(0.22)	-7.18	0.00

*** p<0.01, ** p<0.05, * p<0.1. SS = Sedentary or sleep, LPA = Light physical activity, MPA = Moderate physical activity, VPA = Vigorous physical activity.

† Likelihood ratio test for contribution of relative intensity composition
