# Supplementary material for: Joint associations between objectively measured physical activity volume and intensity with body fatness: the Fenland study
Source: Int J Obes (Lond). 2021 Sep 30;46(1):169–77. doi: 10.1038/s41366-021-00970-8 (PMC8748201; doi:10.1038/s41366-021-00970-8)
Supplement: Supplementary file 12 — Supplemental Table 7 [file 41366_2021_970_MOESM12_ESM.pdf]

Supplementary Table 7: Relationship between isocaloric z1 ILR coordinate and body fat percentage by tertile of PAEE

Women	Tertile 1 (n = 2050)				Tertile 2 (n = 2049)				Tertile 3 (n = 2049)			
	coef	se	tstat	pval	coef	se	tstat	pval	coef	se	tstat	pval
SS:remaining behaviours	-0.07***	(0.02)	-3.41	0.00	-0.26	(0.23)	-1.13	0.26	-0.38*	(0.21)	-1.77	0.08
LPA:remaining behaviours	1.25***	(0.24)	5.11	0.00	0.78***	(0.26)	3.00	0.00	0.05	(0.26)	0.19	0.85
MPA:remaining behaviours	-0.04	(0.14)	-0.28	0.78	-0.08	(0.22)	-0.36	0.72	0.87***	(0.26)	3.35	0.00
VPA:remaining behaviours	-0.41***	(0.06)	-6.85	0.00	-0.45***	(0.05)	-8.53	0.00	-0.55***	(0.06)	-9.92	0.00
Total PAEE	-0.25***	(0.03)	-7.68	0.00	-0.10***	(0.04)	-2.74	0.01	-0.13***	(0.01)	-8.75	0.00
**Men**	**Tertile 1 (n = 1774)**				**Tertile 2 (n = 1773)**				**Tertile 3 (n = 1773)**			
	coef	se	tstat	pval	coef	se	tstat	pval	coef	se	tstat	pval
SS:remaining behaviours	-0.07	(0.19)	-0.36	0.72	-0.35*	(0.20)	-1.71	0.09	-1.28***	(0.19)	-6.67	0.00
LPA:remaining behaviours	0.34	(0.24)	1.43	0.15	1.04***	(0.24)	4.27	0.00	1.47***	(0.23)	6.38	0.00
MPA:remaining behaviours	0.09	(0.17)	0.53	0.59	-0.25	(0.23)	-1.09	0.28	0.15	(0.26)	0.56	0.57
VPA:remaining behaviours	-0.36***	(0.05)	-7.61	0.00	-0.44***	(0.05)	-8.79	0.00	-0.34***	(0.06)	-6.10	0.00
Total PAEE	-0.09***	(0.03)	-3.72	0.00	-0.06**	(0.03)	-2.27	0.02	-0.09***	(0.01)	-6.89	0.00

Note: Beta coefficients indicate direction and significance, but not magnitude of reallocation effect. SS = Sedentary or sleep, LPA = Light physical activity, MPA = Moderate physical activity, VPA = Vigorous physical activity.
*** p<0.01, ** p<0.05, * p<0.1
