# Supplementary material for: Joint associations between objectively measured physical activity volume and intensity with body fatness: the Fenland study
Source: Int J Obes (Lond). 2021 Sep 30;46(1):169–77. doi: 10.1038/s41366-021-00970-8 (PMC8748201; doi:10.1038/s41366-021-00970-8)
Supplement: Supplementary file 13 — Supplemental Table 8 [file 41366_2021_970_MOESM13_ESM.pdf]

Supplementary Table 8: Isotemporal substitution of physical activity and body fat percentage

Women n = 6148	Substituted from SS	Substituted from LPA	Substituted from MPA	Substituted from VPA
Substituted to SS (min/day)	*Dropped*	0.01***<br>(0.01 - 0.01)	0.03***<br>(0.03 - 0.03)	0.22***<br>(0.20 - 0.25)
Substituted to LPA (min/day)	-0.01***<br>(-0.01 - -0.01)	*Dropped*	0.02***<br>(0.02 - 0.02)	0.21***<br>(0.19 - 0.24)
Substituted to MPA (min/day)	-0.03***<br>(-0.03 - -0.03)	-0.02***<br>(-0.02 - -0.02)	*Dropped*	0.19***<br>(0.17 - 0.22)
Substituted to VPA (min/day)	-0.22***<br>(-0.25 - -0.20)	-0.21***<br>(-0.24 - -0.19)	-0.19***<br>(-0.22 - -0.17)	*Dropped*

Men n = 5320	Substituted from SS	Substituted from LPA	Substituted from MPA	Substituted from VPA
Substituted to SS (min/day)	*Dropped*	-0.00***<br>(-0.00 - -0.00)	0.02***<br>(0.01 - 0.02)	0.12***<br>(0.10 - 0.13)
Substituted to LPA (min/day)	0.00***<br>(0.00 - 0.00)	*Dropped*	0.02***<br>(0.02 - 0.02)	0.12***<br>(0.11 - 0.13)
Substituted to MPA (min/day)	-0.02***<br>(-0.02 - -0.01)	-0.02***<br>(-0.02 - -0.02)	*Dropped*	0.10***<br>(0.09 - 0.12)
Substituted to VPA (min/day)	-0.12***<br>(-0.13 - -0.10)	-0.12***<br>(-0.13 - -0.11)	-0.10***<br>(-0.12 - -0.09)	*Dropped*

Note: Data are beta coefficients (95% c.i.). The unit for substitution results is difference in body fat percentage per minute per day substituted. SS = Sedentary or sleep, LPA = Light physical activity, MPA = Moderate physical activity, VPA = Vigorous physical activity.

*** $p<0.01$, ** $p<0.05$, * $p<0.1$
