# Supplementary material for: Joint associations between objectively measured physical activity volume and intensity with body fatness: the Fenland study
Source: Int J Obes (Lond). 2021 Sep 30;46(1):169–77. doi: 10.1038/s41366-021-00970-8 (PMC8748201; doi:10.1038/s41366-021-00970-8)
Supplement: Supplementary file 14 — Supplemental Table 9 [file 41366_2021_970_MOESM14_ESM.pdf]

Supplementary Table 9: Relationship between isotemporal z1 ILR coordinate and body fat percentage

Women n = 6148	coef	se	tstat	pval
SS:remaining behaviours	1.81***	(0.14)	12.94	0.00
LPA:remaining behaviours	-0.45***	(0.17)	-2.66	0.01
MPA:remaining behaviours	-0.90***	(0.08)	-10.99	0.00
VPA:remaining behaviours	-0.46***	(0.02)	-18.59	0.00

Men n = 5320	coef	se	tstat	pval
SS:remaining behaviours	0.40***	(0.14)	2.94	0.00
LPA:remaining behaviours	0.93***	(0.15)	6.12	0.00
MPA:remaining behaviours	-0.99***	(0.09)	-11.28	0.00
VPA:remaining behaviours	-0.34***	(0.02)	-14.34	0.00

Note: Beta coefficients speak to direction and significance, but not magnitude of reallocation effect. SS = Sedentary or sleep, LPA = Light physical activity, MPA = Moderate physical activity, VPA = Vigorous physical activity.
*** p<0.01, ** p<0.05, * p<0.1
