# Supplementary material for: Joint associations between objectively measured physical activity volume and intensity with body fatness: the Fenland study
Source: Int J Obes (Lond). 2021 Sep 30;46(1):169–77. doi: 10.1038/s41366-021-00970-8 (PMC8748201; doi:10.1038/s41366-021-00970-8)
Supplement: Supplementary file 15 — Supplemental Table 10 [file 41366_2021_970_MOESM15_ESM.pdf]

Supplementary Table 10: Relationship between isotemporal pairwise ILR coordinates and body fat percentage

	Women n = 6148				Men = 5320			
	coef	se	tstat	pval	coef	se	tstat	pval
**Composition of intensity (Full ILR coordinate set)**	-	-	-	0.00†	-	-	-	0.00†
**Pairwise reallocation - SS to LPA**								
$ilr_1 \propto$ ln(LPA & SS:MPA & VPA)	2.04***	(0.11)	18.24	0.00	2.00***	(0.12)	16.43	0.00
$ilr_2 \propto$ ln(LPA:SS)	-2.39***	(0.32)	-7.51	0.00	0.56*	(0.29)	1.92	0.05
$ilr_3 \propto$ ln(MPA:VPA)	-0.47***	(0.10)	-4.67	0.00	-0.68***	(0.11)	-6.47	0.00
**Pairwise reallocation - SS to MPA**								
$ilr_1 \propto$ ln(MPA & SS:LPA & VPA)	1.35***	(0.26)	5.25	0.00	-0.88***	(0.23)	-3.75	0.00
$ilr_2 \propto$ ln(MPA:SS)	-2.87***	(0.16)	-17.91	0.00	-1.47***	(0.18)	-8.32	0.00
$ilr_3 \propto$ ln(LPA:VPA)	0.01	(0.18)	0.05	0.96	1.35***	(0.16)	8.43	0.00
**Pairwise reallocation - SS to VPA**								
$ilr_1 \propto$ ln(VPA & SS:MPA & LPA)	2.03***	(0.21)	9.56	0.00	0.08	(0.21)	0.41	0.68
$ilr_2 \propto$ ln(VPA:SS)	-2.40***	(0.15)	-15.91	0.00	-0.79***	(0.15)	-5.34	0.00
$ilr_3 \propto$ ln(MPA:LPA)	-0.48**	(0.24)	-2.03	0.04	-2.04***	(0.22)	-9.27	0.00
**Pairwise reallocation - LPA to MPA**								
$ilr_1 \propto$ ln(MPA & LPA:SS & VPA)	-2.03***	(0.21)	-9.56	0.00	-0.08	(0.21)	-0.41	0.68
$ilr_2 \propto$ ln(MPA:LPA)	-0.48**	(0.24)	-2.03	0.04	-2.04***	(0.22)	-9.27	0.00
$ilr_3 \propto$ ln(SS:VPA)	2.40***	(0.15)	15.91	0.00	0.79***	(0.15)	5.34	0.00
**Pairwise reallocation - LPA to VPA**								
$ilr_1 \propto$ ln(VPA & LPA:SS & MPA)	-1.35***	(0.26)	-5.25	0.00	0.88***	(0.23)	3.75	0.00
$ilr_2 \propto$ ln(VPA:LPA)	-0.01	(0.18)	-0.05	0.96	-1.35***	(0.16)	-8.43	0.00
$ilr_3 \propto$ ln(SS:MPA)	2.87***	(0.16)	17.91	0.00	1.47***	(0.18)	8.32	0.00
**Pairwise reallocation - MPA to VPA**								
$ilr_1 \propto$ ln(MPA & VPA:SS & LPA)	-2.04***	(0.11)	-18.24	0.00	-2.00***	(0.12)	-16.43	0.00
$ilr_2 \propto$ ln(VPA:MPA)	0.47***	(0.10)	4.67	0.00	0.68***	(0.11)	6.47	0.00
$ilr_3 \propto$ ln(SS:LPA)	2.39***	(0.32)	7.51	0.00	-0.56*	(0.29)	-1.92	0.05

*** $p<0.01$, ** $p<0.05$, * $p<0.1$. SS = Sedentary or sleep, LPA = Light physical activity, MPA = Moderate physical activity, VPA = Vigorous physical activity.

† Likelihood ratio test for contribution of relative intensity composition
